# High-performance gas measurement systems—bridging the gap between sensors and analytics

**DOI:** 10.1007/s00216-026-06675-z

**Published:** 2026-07-20

**Authors:** Andreas Schütze, Tilman Sauerwald, Christian Bur

**Affiliations:** 1 Lab for Measurement Technology, Saarland University, Saarbrücken, Germany; 2 Fraunhofer Institute for Process Engineering and Packaging IVV, Freising, Germany

**Keywords:** Gas measurement systems, High-performance, Calibration, Digitization, Machine learning

## Abstract

In gas measurements, there is inherent competition between sensors and analytical systems, often made difficult by a lack of understanding of approaches and constraints from both sides. This leads to controversies, sometimes simple to avoid or defuse, but sometimes leading to harsh discussions and resentment of “wrong” concepts from the respective other side. Meanwhile, collaboration between disciplines is often a key to scientific and, in the long term, economic and societal success. This is especially true today, when digitization is the pervasive topic in our society and where “sensors act as a bridge between biology and digitization.” This article addresses this conflict for the field of high-performance gas measurement systems, where the two worlds of low-cost sensor systems on the one hand and high-performance analytical instruments on the other are still often at odds.

## Introduction

In gas measurements, there is inherent competition between sensors and analytical systems, often made difficult by a lack of understanding of approaches and constraints from both sides. This leads to controversies, sometimes simple to avoid or defuse, but sometimes leading to harsh discussions and resentment of “wrong” concepts from the respective other side. Meanwhile, collaboration between disciplines is often a key to scientific and, in the long term, economic and societal success. This is especially true today, when digitization is the pervasive topic in our society and where “sensors act as a bridge between biology and digitization” [1]. This article addresses this conflict for the field of high-performance gas measurement systems, where the two worlds of low-cost sensor systems (e.g., based on metal oxide semiconductor (MOS) gas sensors [2], often referred to as electronic nose [3–5] or E-nose after the first description of the concept by Persaud and Dodd [6]) on the one hand and high-performance analytical instruments (e.g., the gold standard gas chromatography-mass spectrometry (GC-MS) [7]) on the other are still often at odds. An indication of this point can be found in many examples, with the Air Quality directive of the European Union of 2008 as a striking example [8]. In this, as for many other cases, analytical tools and measurements are demanded for obtaining official data. Due to the trend of low-cost sensors, especially for smart cities and citizen science applications, the air quality directive also allowed measurements using sensors; however, those were by default assumed to be of lower quality and could only be used for so-called indicative measurements with higher uncertainties. While it is obvious that the uncertainty of each single sensor node increases when being calibrated against a reference instrument, the question remains how to best estimate the quality of air. A very sparse grid of analytical reference stations might have high accuracy at the sampling point but does not represent air quality well in between the stations. Using a dense sampling grid of low-cost sensor nodes, each with potentially higher uncertainty, could outweigh the disadvantages and lead to a better representation of the overall air quality. This facilitates a better insight into personal expositions and potential mitigation strategies with a higher spatial resolution. For example, the concentration of hazardous gases in urban air can vary from street to street or even depends on the side of the street which is known as the street canyon effect [9, 10]. The key to solving the complex application of air quality monitoring lies mostly in combination of some analytical reference stations and a high-performance, low-cost sensor network. For other use cases, like Indoor Air Quality (IAQ), the use of only high-performance sensor systems seems sufficient. The goal is always to provide an application-oriented solution allowing for reliable measurements with reproducible and traceable results.

## High performance at low-cost

**Fig. 1:**
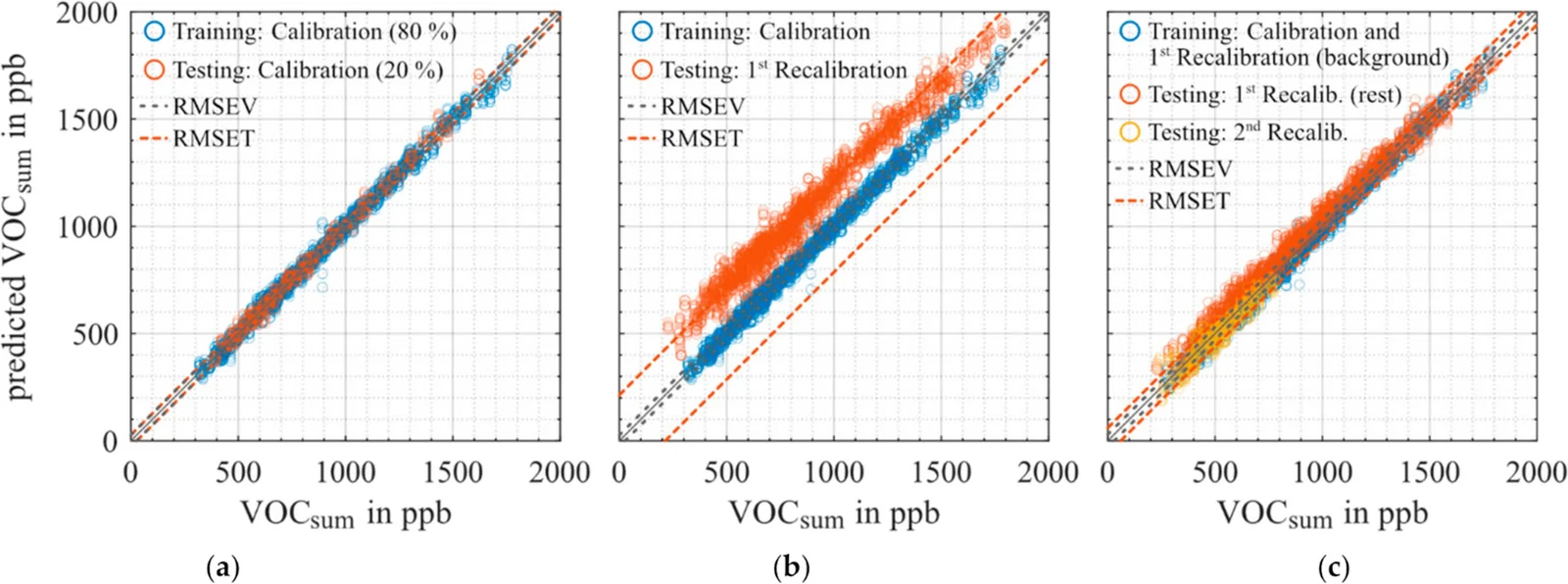
Example for high-performance gas sensor systems for Indoor Air Quality (IAQ) monitoring. The sum concentration of all volatiles is desired for ventilation control to detect the presence of persons in a room as well as other contaminations from human activities (cleaning, cooking, etc.) as well as building materials, furniture etc. **a** Initial calibration shows high performance with low root mean square error (RMSE) for both validation (V) and test (T) data. **b** After several weeks of field operation, drift is observed by the shift of data recorded in a 1^st^ recalibration campaign. **c** This drift can be largely eliminated by extending the calibration leading to negligible drift for the 2^nd^ recalibration after further extended field operation [29]

**Fig. 2:**
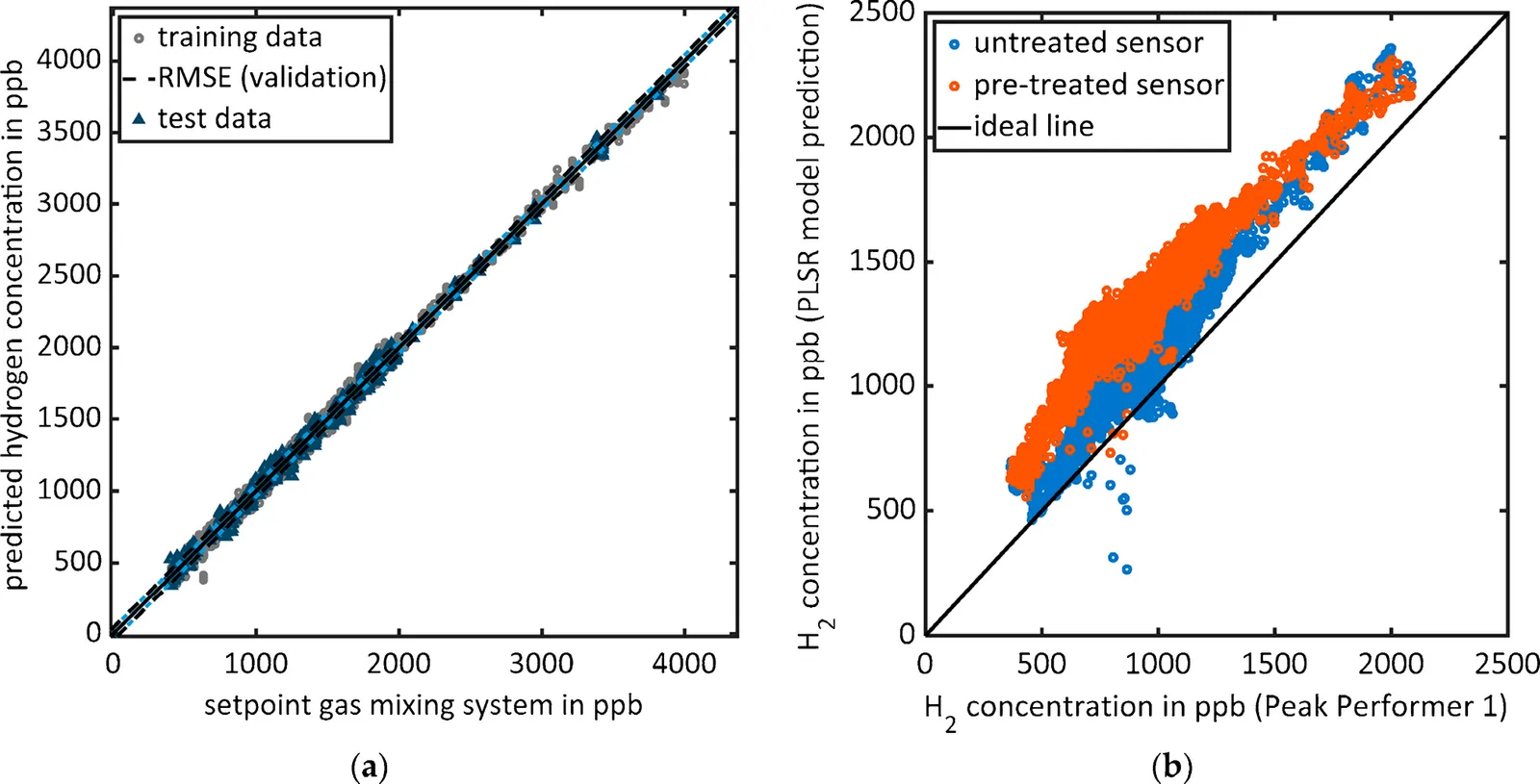
**a** Calibration curve for hydrogen based on a single MEMS gas sensor with dynamic operation; the calibration also considers changes of RH as well as interfering gases (CO, ethanol, acetone, toluene, and formaldehyde in the ppm range). The RMSE for validation as well as test data with unknown gas mixtures is < 45 ppb. **b** Comparison of sensor data recorded in the field over several days with a GC with reducing compound photometer (RCP) detector (Peak Performer 1, Peak Laboratories Inc., Mountain View, CA, USA) as analytical reference. Note that the current atmospheric background concentration of hydrogen is approx. 530 ppb [30]; thus, the offset between sensor and reference is mostly due to the GC which shows values down to approx. 400 ppb. [26]

However, the example of Indoor Air Quality (IAQ) proves that high-performance can be achieved with sensor systems based on low-cost MEMS devices [11] in calibration as well as in field experiments, see Figs. 1 and 2, respectively, if the quality along all steps of the development chain is assured [12]. This performance is based on making use of the full potential of digitisation for sensors systems: These are based on highly miniaturised and integrated micro-electro-mechanical system (MEMS) sensors, today combining multiple sensing layers (often four) with micro-hotplates for low power consumption and integrated electronics for sensor operation and read-out [13, 14]. The second building block is based on dynamic operation of the sensors, allowing them to record rich raw data from the sensor, e.g., by temperature cycling, impedance spectroscopy, or optical excitation [15, 16]. These high-dimensional data patterns are interpreted with machine learning, which of course requires suitable calibration approaches. These can be based on reference measurements, as for example suggested for sensor modules for outdoor air quality monitoring [17, 18], which of course cannot achieve higher performance than these reference measurements. Alternatively, laboratory calibration can be employed, which however requires close simulation of ambient conditions in the later application. Very high accuracy can be achieved using complex gas mixing systems [19–21], advanced calibration strategies [22–26] and rigorous validation in the lab and in the field [21, 26–28].

Long-term stability can also be addressed through various drift compensation methods. One proven approach is based on extended calibration, i.e., by building a calibration model that is based on data recorded over a longer period of time in the field and thus containing drift occurring in the later application. The ML model is thus required to suppress drift effects and achieves stable operation over extended periods of at least several months, cf. Figure 1 [29].

This example also shows where reservations and misunderstanding between analytical and sensor communities arise: In both communities, a non-targeted approach is aiming at a direct correlation of a state (e.g., the odour of a product or the health of a human being) to a measurement of chemical properties without predefining certain targets that are thought to be comprehensive descriptors of the state. Non-targeted analytical measurements are aiming at a complete understanding and traceability of each chemical compound in the sample. Ideally, each compound is identified, meaning that they have been tested with a reference substance; in any case, compounds are detected by mass spectrum and retention index. This enables traceability of all measurands and reproducibility of the method. The results can be used to fully understand the relation of the state with the composition of chemicals and ideally to define the contributing compounds as targets. Following targeted analytics, limited to compounds that are already well understood, can then be used to measure high numbers of samples with reduced effort. In non-targeted sensor measurements, patterns of multisensory arrays are directly correlated with certain states. It is usually impossible to explain the relation between the sensor output and the chemical composition of the state. This setup cannot be traced to SI units, and in most cases, reproducibility is very limited, and the results are rarely transferable to another device. A sensible way of training sensor systems is, in our opinion, always based on a full understanding of the chemical composition of the application and a respective (and traceable) training.

It requires a comprehensive effort to sample, detect, and identify all compounds in a non-targeted analytical approach. Therefore, for practical reasons, even very comprehensive non-targeted approaches may neglect certain compounds that may affect the sensor system (for example, very volatile compounds are often not sampled by sorption traps). The neglected substances might not even be close to target compounds but could still limit the comparability of analytical and sensor results (e.g., reducing permanent gases like NO and ozone affecting the sensor signals). Even a comprehensive understanding of the application may contain certain unknown ambient conditions which may lead to false predictions by an insufficiently trained sensor system [31].

However, to achieve the highest quality, the sensor system would also have to be adapted to the application scenario and calibrated under the true operating conditions. To calibrate sensor systems for a given application, it is important to identify target and interfering substances representing the application. Typically, analytical methods are used here which enable and facilitate the development of data-driven models of a sensor system. For the Indoor Air Quality (IAQ) example in Figs. 1 and 2, the sensors were calibrated and trained on a representative database which includes substances identified by analytical screening measurements. The sensor systems were lab calibrated with randomly distributed VOC mixtures including permanent gases and non-organic compounds. The discovery of confounding factors without analytical measurements seems impossible.

Machine learning for sensor systems is always limited to the defined calibration space: situations not represented in the calibration, i.e., new gases, other mixtures, and concentrations not contained in the calibration, can in general not be interpreted correctly. In other words, ML methods typically fail in extrapolating. This is often seen as severe limitation from the perspective of analytical chemistry and is even exacerbated by many published gas sensor studies which are based on very limited or even no calibration purely taking the datasheet parameters as truth, which is simply not acceptable from a scientific perspective. The most often stated reason for taking the datasheet values is the high cost associated with calibration when following rigorous standards, which is seen as contradiction to the low-cost nature of the sensors. Indeed, the effort of calibration needs is limiting their use to applications that require a high number of systems. A stronger collaboration between analytical sciences and sensor technology could mitigate this problem and would be highly beneficial for both sides as both can ideally complement each other. An integration of analytical measurement and sensor testing for example could reduce the effort of sample preparation and data processing by providing synchronized signals for the same sample [32, 33]. In terms of methods, for example round robin tests that are widely accepted to ensure quality and identify problems in analytics should be implemented in the sensor community, where it would mean testing sensor systems in different labs with different test systems (cf. [30, 34]). Inversely, sensor systems are irreplaceable whenever continuous monitoring with high spatial and temporal resolution is required, which is ideal to identify hotspots for in-depth analytical measurement. This could be used to develop better sampling strategies for analytics, which is probably the most underestimated problem of analytical measurements. On the other hand, sensor systems cannot interpret unknown or novel situations, but these can be revealed by performant ML models using novelty detection [35–37]. In this case, a complementary sampling could be performed for analytical interpretation and quantification of the situation, which could then be used to update the sensors ML model. Furthermore, novelties could lead to hidden chemical parameters (a class chemical compound, e.g., very volatile compounds, which have not been assessed by the analytics, but might be important to describe the application). This can be used for the detection of “blind spots” in a non-targeted analytical approach.

### Application specific performance requirements

Note that for some studies the high spatial resolution of gas sensor systems can be leveraged to achieve valuable insights even without quantitative accuracy as long as the sensor signal is monotonically dependent on the target gas concentration and a proper design of experiment is used. For example, a study with mobile sensor systems in central Copenhagen revealed significant differences of NO_2_ exposure for pedestrians and joggers along two paths which were only a few m apart [38]. In general, many modern “digital” applications do not require high-performance quantification, e.g., the US AQI index for reporting outdoor air quality (https://www.airnow.gov/aqi/aqi-basics/) as the basis for information displayed on Google maps. Similarly, ventilation control in smart homes also requires only rough estimates, but on a continuous basis with high temporal resolution.

### Machine learning and virtual sensors

Note that in this context the term *virtual sensors* is often used, which is mostly interpreted as a sensor not measuring the targeted quantity directly: a *virtual sensor* is a pure software sensor (therefore also called *soft sensor*) which autonomously produces signals by combining and aggregating signals that it receives from physical or other virtual sensors [39]. However, if this definition is taken seriously, then not only sensor systems based on ML fall into this category, but many widely used sensors do as well: most flow sensors are based on pressure drop or thermal transport or Coriolis force, so none measures flow directly. A more appropriate differentiation seems to be if the data interpretation is based on physical modelling or on data-based models, i.e., machine learning. Note however, that also physical-model-based sensors typically require calibration; i.e., the physical model is supported by data. This trend has been existing in sensor technology for many years now and is widely accepted especially in the context of the Internet of Things (IoT) or, more generally, digitization as driving developments such as Industrie 4.0 [40]. In gas sensing, automatic baseline correction (ABC) for CO_2_ sensors is an illustrative example for this approach (https://senseair.com/the-senseair-abc-algorithm/): it assumes that the lowest recorded value over a longer period (defined by the manufacturer) corresponds to the atmospheric background concentration and is used as one-point calibration. In the example of IAQ monitoring presented above, the high performance is again achieved based on in-depth understanding of processes on the sensor surface as a basis for sensor operation, comprehensive calibration and data analysis based on gas-specific reaction rate constants [41].

## Combining analytics and sensors opens new applications

**Fig. 3:**
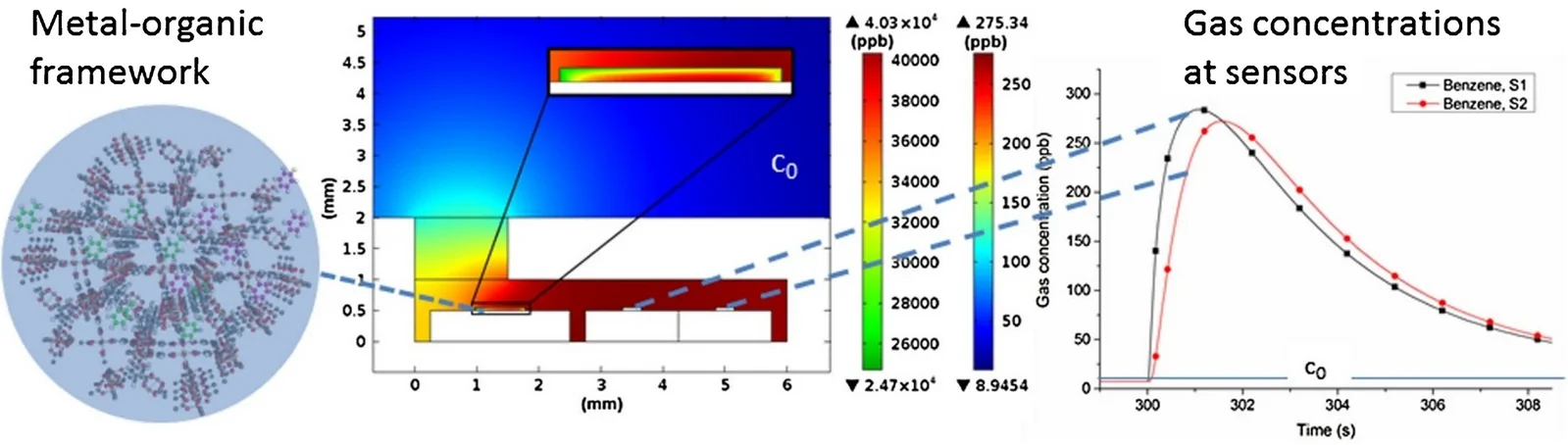
A highly miniaturized integrated gas sensor system combining a pre-concentrator with MOF layer from analytical science with dynamic operation from sensor systems to achieve improved sensitivity and selectivity; the approach is based on gas diffusion avoiding the requirement of valves or pumps (graphical abstract from [42])

**Fig. 4:**
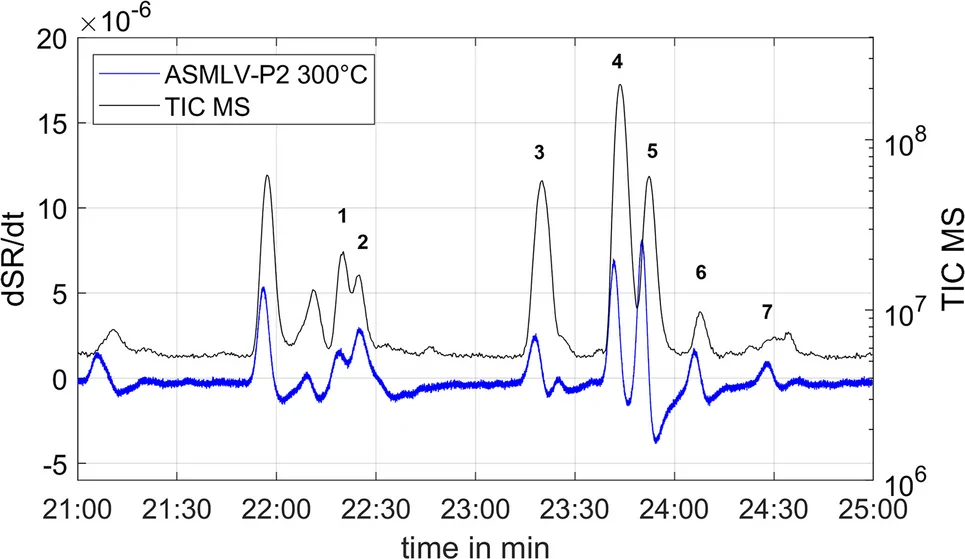
Comparison of chromatograms recorded with an MS and a MOS gas sensor for a complex [44]. Note that the gas sensor signal shows the time derivative to compensate for the slow recovery of the sensor. The gas sensor shows much better SNR than the MS for 4-Chlortoluene (peak 7). For both detectors, accurate identification and quantification require suitable calibration

Close interaction between analytical and sensor sciences not only improves mutual understanding but also allows addressing applications that are beyond either approach today. For example, incorporating analytical methods such as preconcentration in gas sensor systems allows reducing the detection limit and at the same time improving selectivity of gas sensor systems as depicted in Fig. 3 [42]. The preconcentration step can be compared to dynamic headspace sampling in chemical analytics and, obviously, a precontraction step the dynamic of the systems (300 s sampling time in the given example) and the additional hotplate adds to the total power consumption. A rigorous miniaturization strategy is necessary to balance the trade off between additional performance and costs. Making use of the high sensitivity of MOS gas sensors as detectors for GC systems combines high selectivity and low cost allowing new applications for portable analytical systems or in-process quality control [32, 33, 43, 44], cf. Figure 4.

Applications requiring the combination of features from analytics and sensors, i.e., continuous measurements with high specificity, or simply the close collaboration in R&D between analytical measurements and sensor systems are widespread. High-performance Indoor Air Quality (IAQ) monitoring does not only allow demand-controlled ventilation, but also scientific studies such as the impact of air pollution on cognitive functions [45] or on the role of specific VOCs for health and well-being [46]. Food safety and reduced food waste offer great potential both in households and along the supply chain [47–49]. The holy grail for gas sensor systems for many years has been cancer screening [50–55], but simple non-targeted analysis, i.e., blind discrimination between healthy and cancer patients, without analytical ground truth will not be successful, while drug monitoring with known target and interfering gases shows very promising results [56]. Combining the strengths of both fields even allows us to efficiently solve exotic applications, i.e., for sustainable conservation of historic artefacts in museum display cases [57, 58].

## Outlook

Pervasive digitisation combining miniaturised sensors and machine learning is an ongoing trend that will open new insights not only for gas measurements. Based on these insights, new applications that require fast, inexpensive, mobile, and/or distributed measurement systems will become accessible., is an ongoing trend that not only will open new insights into gas measurements but also will lead to new applications that require fast, inexpensive, mobile, and/or distributed measurement systems. Even if the understanding of sensor values might be limited at first, there is a strong driving force to make use of such systems, gathering data which might later be better interpretable. However, neglecting basic principles of data quality, first and foremost by comprehensive calibration, but also in providing metadata according to the FAIR principles [61] will prevent making sense of collected (big) data. This holds not only for MEMS and ML but also for further new trends such as quantum sensing [59]. On the other hand, good analytical understanding of the scenarios can facilitate the understanding of novel sensor approaches and ensure the integrity of results. It will be important how this knowledge can be transformed into new measurements and calibration standards [60]. This needs to be complemented by standardised sensor network protocols and data interfaces. Especially crucial is the exchange standard of metadata, e.g., temperature operation protocols, which are crucial for reproducibility and comparability of the results, which is a consistent application of the FAIR principles (reusable). This gives the most comprehensive insight into reality and will provide analytical investigations with the most promising questions, validation tasks, and challenges. In this sense, a constructive interaction between analytical and sensor expertise in the field of gas measurement systems would stimulate both fields and advance science and also society as a whole.
